# The endometriosis daily diary: qualitative research to explore the patient experience of endometriosis and inform the development of a patient-reported outcome (PRO) for endometriosis-related pain

**DOI:** 10.1186/s41687-021-00409-8

**Published:** 2022-01-15

**Authors:** Yanfen Guan, Allison M. Nguyen, Samantha Wratten, Sharan Randhawa, Jessica Weaver, Felipe Arbelaez, Arnaud Fauconnier, Charlotte Panter

**Affiliations:** 1 Merck Research Laboratories, 2000 Galloping Hill Road, Kenilworth, NJ 07033 USA; 2 Adelphi Values, Adelphi Mill, Bollington, Cheshire, SK10 5JB UK; 3 CHI Poissy-St-Germain, Service De Gynécologie & Obstétrique, 10 rue du champ Gaillard, BP 3082, 78303 Poissy CEDEX, France

**Keywords:** Endometriosis, Patient-reported outcomes (PROs), Development, Qualitative, Content validity, Endometriosis-related pain (ERP)

## Abstract

**Purpose:**

Endometriosis is a chronic disorder of the female reproductive system characterized by debilitating symptoms, particularly endometriosis-related pain (ERP). Patient-reported outcome (PRO) measures of symptoms and impacts are required to assess disease severity in ERP clinical studies and clinical practice. A content-valid instrument was developed by modifying the Dysmenorrhea Daily Diary (DysDD) to form the Endometriosis Daily Diary (EDD), an electronic PRO administered via handheld device.

**Methods:**

Qualitative research with US females with ERP was conducted in three stages: (1) Development of an endometriosis conceptual model based on qualitative literature and conduct of concept elicitation (CE) interviews (N = 30). (2) Cognitive debriefing (CD) interviews (N = 30) conducted across two rounds to assess relevance and understanding of the EDD, with modifications between interview rounds. (3) Pilot testing to assess usability/feasibility of administrating the EDD daily on an electronic handheld device (N = 15). Clinical experts provided guidance throughout the study.

**Results:**

The conceptual model provided a comprehensive summary of endometriosis to inform modifications to the DysDD, forming the EDD. CD results demonstrated that EDD items were relevant for most participants. Instructions, items, response scales, and recall period were well-understood. The resulting daily diary assesses severity of cyclic and non-cyclic pelvic pain, dyspareunia, impact of ERP on functioning and daily life, symptoms associated with ERP, and bowel symptoms. Participants were able to complete the diary daily and found the device easy to use.

**Conclusion:**

The EDD demonstrated good content validity in females experiencing ERP. The next step is to perform psychometric validation in an ERP sample.

**Supplementary Information:**

The online version contains supplementary material available at 10.1186/s41687-021-00409-8.

## Background

Endometriosis is a chronic, progressive disease characterized by the presence of endometrial tissue outside the uterine cavity, causing irritation, bleeding, and inflammation in the ectopic tissue. Worldwide prevalence is estimated at 6–10% of women of reproductive age [1, 2]. The most frequently mentioned symptoms include severe dysmenorrhea (painful menstruation), non-menstrual pelvic pain, pain with sexual intercourse (dyspareunia), heavy menstrual bleeding and/or spotting between periods [3, 4]. As such, endometriosis can have a substantial impact on patients’ quality of life [5–7]. Undergoing a surgical procedure (e.g., laparoscopy) can both confirm diagnosis and treat the disease, however, such invasive procedures are often only initiated after failure of therapeutic interventions (e.g. hormonal medication) [8]. Thus, endometriosis patients face significant delays in diagnosis which can prolong suffering [8, 9]. Without surgical intervention, one of the primary ways of determining disease severity is based on patient descriptions of their symptoms and how they impact their lives using patient-reported outcomes (PROs).

PROs can assess clinical trial endpoints for new therapies [10], alongside other clinical endpoints. An in-depth literature review and instrument review was conducted to identify PRO instruments that assess key symptoms and impact of endometriosis-related pain (ERP) in clinical trials. The findings suggested that historically, the most commonly used instrument for assessing endometriosis symptoms in a clinical setting was the clinician-completed Biberoglu and Behrman (B&B) scale [11], although the instrument is subject to potential rater bias and there is no documented evidence of patient input in the development process, which is an integral component for instruments being used in clinical trials for supporting regulatory claims [12]. A number of endometriosis PRO instruments have been developed in accordance with regulatory and best practice guidelines [10, 13] to overcome these limitations, including the Endometriosis Pain Daily Diary (EPDD), the Endometriosis Symptom Diary (ESD), and the Endometriosis Impact Scale (EIS) [14, 15]. However, at the time when this work was initiated, the psychometric properties of EPDD had not yet been established according to a review of the published literature, and EID and ESD development work had not been published and the instruments were not available for review due to the proprietary nature. As such, there was a need to develop a fit-for-purpose instrument to be used in the clinical development program of a novel ERP treatment.

The Dysmenorrhea Daily Diary (DysDD) [16, 17] is a PRO measure that has been developed to regulatory standards [12, 18] for use in primary dysmenorrhea clinical trials and has been fully validated in that disease population. The DysDD assesses key symptoms and impacts of dysmenorrhea which are also experienced by patients with ERP including pelvic pain and the associated impact of pelvic pain on functioning/health-related quality of life (HRQoL) (although ERP was not limited to bleeding days) [19]. The first objective of the present study was to assess the content validity of the DysDD in an ERP population through qualitative concept elicitation (CE) interviews and cognitive debriefing (CD) interviews with ERP patients. A qualitative assessment of content validity is the extent to which the instrument measures concepts of interest, and is supported by open-ended patient input from the appropriate target population to ensure the content is comprehensive, relevant, and understandable [12, 20]. A second objective was to identify if any further revisions or additions should be implemented for the instrument to be adequate for use in the context of ERP clinical trials. The newly formed instrument has been named the Endometriosis Daily Diary (EDD), which measures key concepts of interests related to ERP, including the key symptoms of ERP and the impact of ERP on patients’ HRQoL (functioning and daily life).

## Methods

**Fig. 1:**
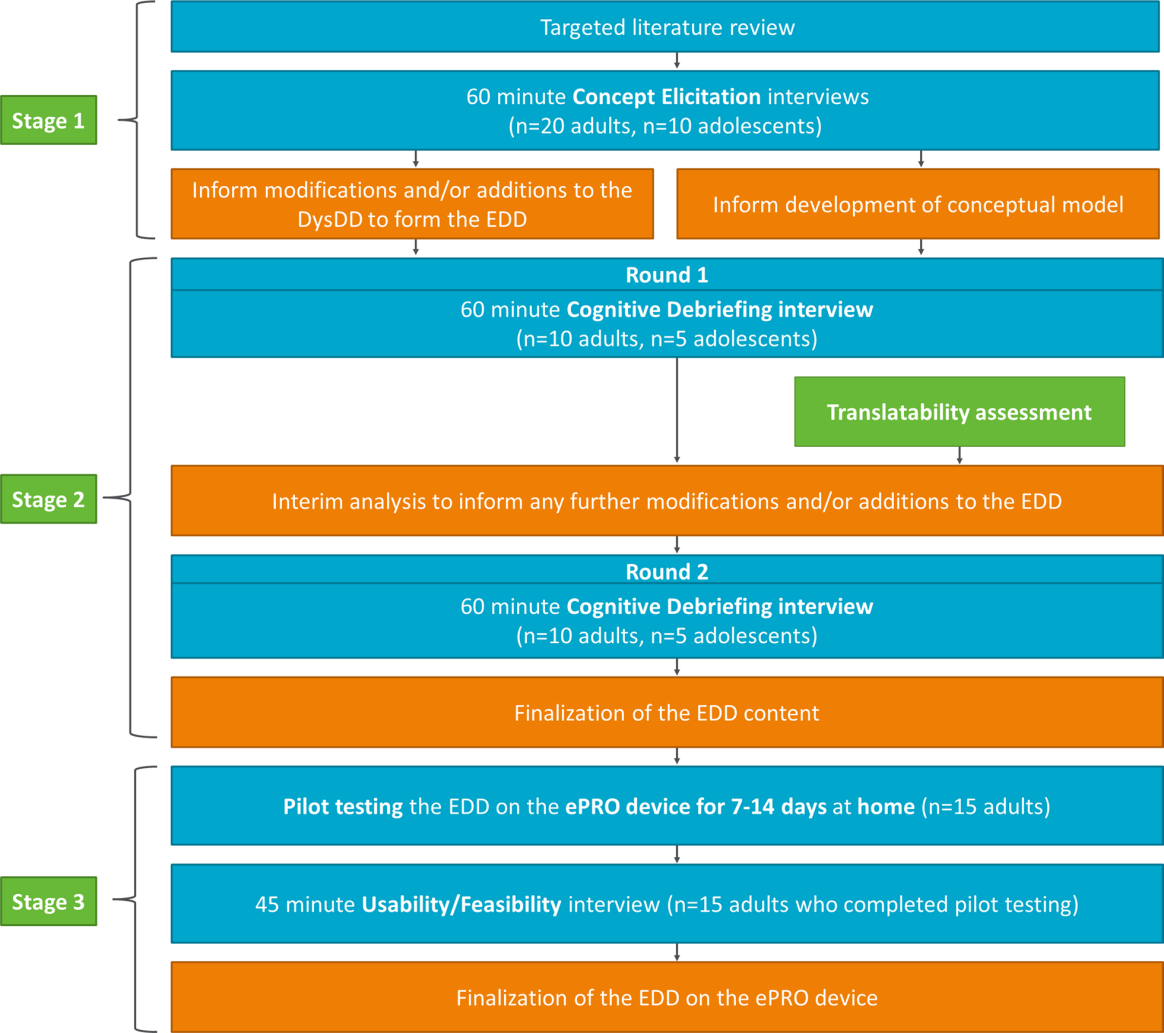
Study design. *Note*: across the samples for the three stages, there was an overlap of five participants, hence a total of 70 independent participants were involved in the study (n = 3 took park in both a CE and a CD interview; n = 2 took part in both a CD interview and a usability/feasibility interview)

This was a qualitative study comprising three key stages to inform the development of the EDD in line with best practice guidelines [10, 13] (Fig. 1): (1) Development of a conceptual model of ERP to inform modifications to the DysDD based on a literature review and CE interviews to understand patient experience of ERP; (2) Two rounds of CD interviews to assess patient understanding, interpretation, and relevance of the newly drafted EDD; (3) Usability and feasibility testing of daily completion of the EDD on an electronic PRO (ePRO) device. Two clinical experts in the field of endometriosis provided input and guidance as research partners at key stages throughout the research.

### Sample

Adolescents and adults with endometriosis were recruited by a recruitment agency via referrals from practicing primary care physicians or gynecologists based in three United States (US) cities: Chicago, Baltimore, and Pittsburgh. All participants spoke US-English, and experienced cyclic (secondary dysmenorrhea) and/or non-cyclic ERP. Eligible adult women were between 18 and 49 years of age inclusive, pre-menopausal, and had a surgical (laparoscopy or laparotomy) diagnosis of endometriosis. Eligible adolescents were at least two years post-menarche and up to 17 years of age inclusive, with either a surgical (laparoscopy or laparotomy) diagnosis of endometriosis or clinically suspected endometriosis (based on presence of pelvic pain incompletely relieved by non-steroidal anti-inflammatory drugs and/or oral contraceptive, ultrasound or other imaging, and/or family history). A quota approach to sampling was employed at each stage to ensure a range of demographic and clinical characteristics.

In qualitative research, sample size is typically determined based on the goal to achieve ‘concept saturation’, a point at which no new concepts are likely to emerge with further interviews [21, 22]. While it is difficult to predict how many interviews are necessary to achieve saturation, research suggests that 10–15 patients will capture > 90% of concepts in a relatively homogeneous population [23]. As such, a minimum of 15 patients were recruited at each stage of the research.

### Procedure

The study was approved and overseen by an Independent Review Board in the US (approval code: 420,180,415). Prior to any data collection, written informed consent was obtained from all adult participants and the parent/guardian of all adolescent participants. Written assent was also obtained from adolescent participants.

All interviews were conducted by experienced female interviewers who were briefed on the objectives of the study. At each stage, interviewers used a semi-structured interview guide to ensure consistent exploration of topics. All interviews were audio-recorded and transcribed verbatim. The specific procedures for each stage are outlined below.

### Stage 1: development of a conceptual model of endometriosis

To understand the patient perspective of endometriosis, a review of published qualitative literature (24 articles and 3 conference abstracts/posters published in English between 2000—2018) and publicly available UK and US online patient forums was conducted, which informed the content of the CE interview guide. Face-to-face CE interviews were conducted with ERP patients to explore their experience of symptoms and the associated impacts. Interviews were 60-min and started with open-ended, exploratory questions designed to facilitate spontaneous, unbiased responses, followed by focused questions if concepts had not been fully explored and where concepts of interest had not emerged spontaneously. A conceptual model was developed and used to map relevant symptom and impact concepts onto the DysDD to assess concept coverage and inform any modifications to form the draft EDD.

### Stage 2: cognitive debriefing and translatability assessment of the newly drafted EDD

The CD interviews aimed to assess the content validity of the newly drafted EDD (the modified DysDD). The CD interviews were 60-min and conducted face-to-face across two rounds to allow any modifications to the EDD to be implemented (based on the first round) and tested in the second round. Participants completed the draft EDD using a ‘think aloud’ approach [24] where they were asked to share their thoughts as they read each instruction/item and selected each response, which helped to identify any aspects that were understood or interpreted incorrectly. Participants were then asked questions about their interpretation and understanding of instructions and item wording, the relevance of concepts, the appropriateness of the response options and recall period.

As the EDD may be used globally in future studies, translation into other languages may be required; as such, a translatability assessment was conducted to inform any item wording modifications that would be needed to maintain conceptual equivalency when translated into other languages [25]. The translatability assessment was conducted in parallel to round one interviews to allow any modifications to be implemented and tested in the second round of interviews.

### Stage 3: usability and feasibility testing of the electronic EDD (pilot testing)

Given the intention to administer the EDD on an electronic handheld device for daily at-home completion, this stage explored the usability of the device and feasibility of daily completion with ERP patients. Participants received training and instructions on how to use the device and how to complete the EDD each day, and then were asked to complete the EDD on the device at-home every evening for 7–14 days. Following this, participants completed a 45-min telephone interview to provide feedback on the training, and the usability and feasibility of the electronic EDD.

### Analysis

All interview transcripts were subject to thematic analysis [26] using ATLAS.ti software (ATLAS.ti Scientific Software Development GmbH; Berlin, Germany). A coding scheme was established by the research team to ensure consistent application and grouping of codes across transcripts. New codes were organically added throughout analysis and previously analysed transcripts were re-analysed to ensure that new codes were applied consistently across all transcripts. To evaluate concept saturation, Stage 1 (CE) transcripts were chronologically grouped into equal sets and concepts emerging from each additional set of interviews were compared [21, 22]. Saturation was deemed achieved when no new concepts emerged in the final set.

## Results

In Stage 1, 30 females (20 adults and 10 adolescents) took part in a CE interview. In Stage 2, 30 females (20 adults and 10 adolescents) took part in a CD interview (conducted across two rounds). Finally, in Stage 3, 15 adult females took part in pilot testing and a usability/feasibility interview. Three participants took part in both the CE interview and the first round of CD interviews, and two participants took part in both a CD interview and the usability/feasibility interviews. There was no further overlap in participants. In total, 70 independent participants were involved in this research.

### Participant characteristics

The demographic and clinical characteristics of each sample across the three stages are summarized in Table 1. Overall, the samples show demographic diversity, with ages ranging from 12 to 49 years old. Across the three stages, almost half of participants were White (37/75 [49.3%]) and nearly a third of participants were Black/African American (24/75 [32.0%]). Further, both Non-Hispanic/Latino (48/75 [64.0%]) and Hispanic/Latino (27/75 [36.0%]) females were represented. There was also variation in participants’ education level and current work status.

**Table 1:** Demographic and clinical characteristics of each sample across stages

Description		Stage 1 (N = 30)	Stage 2 (N = 30)	Stage 3 (N = 15)
Demographic characteristics				
Age (years)	Mean (Min, max)	29.4 (12, 49)	29.2 (12,49)	32.5 (19, 45)
Length of time experiencing pelvic pain, years	Mean (Min–Max)	8.7 (0.5–27)	5.8 (1–16)	7.13 (1- 27)
Race, n (%)	White	14 (46.7%)	14 (46.7%)	9 (60.0%)
	Black/African American	12 (40.0%)	9 (30.0%)	3 (20.0%)
	Other	4 (13.3%)	7 (23.3%)	1 (6.7%)
	North African or Alaska Native	0 (0.0%)	0 (0.0%)	1 (6.7%)
	Multi-Racial	0 (0.0%)	0 (0.0%)	1 (6.7%)
Ethnicity, n (%)	Non-Hispanic or Latino	18 (60.0%)	21 (70.0%)	9 (60.0%)
	Hispanic or Latino	12 (40.0)	9 (30.0%)	6 (40.0%)
Highest level of education (adults only), n (%)	College or university degree	7 (23.3%)	6 (20.0%)	2 (13.3%)
	High school diploma or General Education Diploma	7 (23.3%)	4 (13.3%)	4 (26.7%)
	Some years of college	4 (13.3%)	6 (20.0%)	5 (33.3%)
	Certificate Program	2 (6.7%)	3 (10.0%)	3 (20.0%)
	Graduate or professional degree	0 (0.0%)	1 (6.7%)	1 (6.7%)
Work status, n (%)	Working full or part time	17 (56.7%)	19 (63.3%)	13 (86.7%)
	At school or student	10 (33.3%)	9 (30.0%)	1 (6.7%)
	Full time homemaker	3 (10.0%)	2 (6.7%)	1 (6.7%)
*Clinical characteristics*				
Time since diagnosis*, years	Less than 5 years	15 (50%)	21 (70.0%)	8 (53.3%)
	6–10 years	6 (20%)	3 (10.0%)	6 (40.0%)
	Over 10 years	9 (30.0%)	6 (20.0%)	1 (6.7%)
Type of chronic pelvic pain, n (%)	Cyclic pain only	16 (53.3%)	19 (63.3%)	9 (60.0%)
	Cyclic and non-cyclic pain	14 (46.7%)	11 (36.7%)	6 (40.0%)
Severity of cyclic (menstrual) pelvic pain at screening using 0–10 Numerical Rating Scale (NRS)***, n (%)	Mild (1–4)	7 (23.3%)	6 (20.0%)	5 (33.3%)
	Moderate (5–7)	12 (40.0%)	9 (30.0%)	5 (33.3%)
	Severe (8–10)	11 (36.7%)	15 (50.0%)	5 (33.3%)
	Mean (range)	6.53 (3–10)	7.27 (3–10)	6.07 (2–10)
Severity of non-cyclic (non-menstrual) pelvic pain at screening using 0–10 NRS***, n (%)	Mild (1–4)	10 (33.3%)	12 (40.0%)	4 (26.7%)
	Moderate (5–7)	13 (43.3%)	9 (30.0%)	7 (46.7%)
	Severe (8–10)	7 (23.3%)	9 (30.0%)	4 (26.7%)
	Mean (range)	5.6 (3–10)	6.13 (3–10)	5.87 (2–10)
Diagnostic procedure, n (%)	Surgical diagnosis	30 (100%)	20 (66.7%)	15 (100%)
	Clinically suspected endometriosis	0 (0.0%)	10 (33.3%)	0 (0.0%)
Hormonal therapy, n (%)	On hormonal therapy	15 (50.0%)	15 (50.0%)	9 (60.0%)
	Not on hormonal therapy	15 (50.0%)	15 (50.0%)	6 (40.0%)
Current treatment**, pharmacological (non-hormonal), n (%)	Nonsteroidal Anti-Inflammatory Drugs	25 (83.3%)	24 (80.0%)	12 (80.0%)
	Other pain medications (incl. Acetaminophen)	2 (6.7%)	15 (50.0%)	4 (26.7%)
	Opiate (incl. codeine, oxycodone)	3 (10%)	3 (10.0%)	1 (6.7%)

^*^This has been calculated from date of interview

^**^More than one current/previous treatment was reported for some participants

^***^The following interpretative pain severity cut-offs were employed for the 0–10 NRS: 1–4 was considered Mild, 5–7 was considered Moderate, and 8–10 was considered Severe

Just over half of participants experienced cyclic pain only (44/75 [58.6%]), others experienced both cyclic and non-cyclic pain (31/75 [41.3%]). As per the inclusion criteria, all adult participants had been surgically diagnosed. All adolescents in Stage 1 had been surgically diagnosed and all adolescents in Stage 2 had clinically suspected endometriosis. Regardless, participants had been diagnosed in the last five years (44/75 [58.7%]) and just over half of participants were on hormonal therapy at the time of their participation (39/75 [52.0%]). At screening, participants had mostly rated their cyclic pain in the past month as ‘severe’ (31/75 [41.3%]), and their non-cyclic pain as ‘moderate’ (29/75 [38.7%]).

### Stage 1: development of a conceptual model of endometriosis

**Fig. 2:**
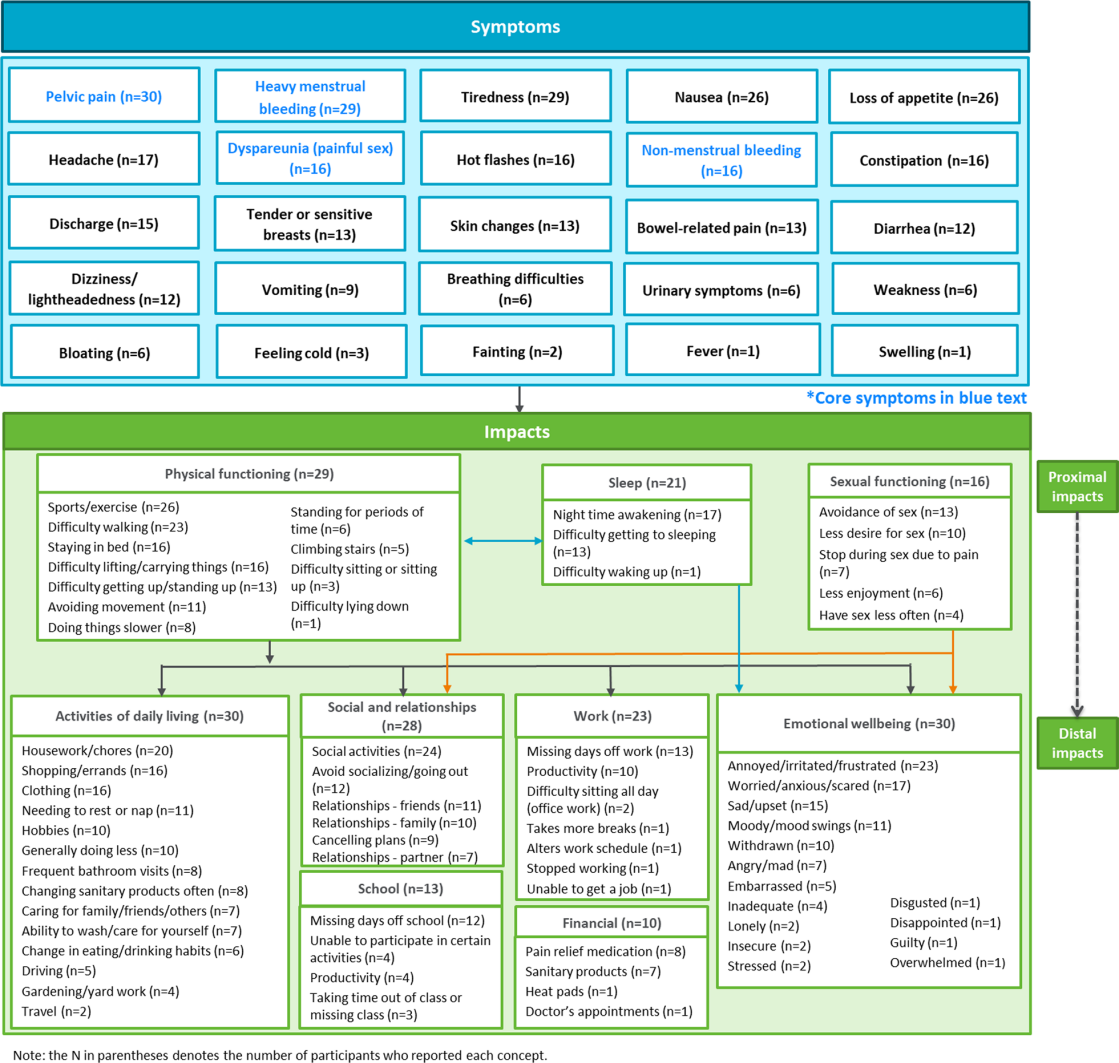
Conceptual model of the experience of endometriosis based on the CE discussion with 30 interview participants

The literature review identified many symptoms and impacts of endometriosis and these helped to inform the development of the semi-structured interview guide used in the CE interviews. During the CE interviews, participants reported 25 distinct symptoms related to their endometriosis (Fig. 2). Core symptoms of endometriosis (shown in blue), as established in the published literature, were reported by the majority of participants: pelvic pain (n = 30/30; [100%]), heavy menstrual bleeding (n = 29/30 [96.7%]), non-menstrual bleeding (n = 16/30 53.3%), and dyspareunia (n = 16/30 [53.3%]). Of note, adolescent participants were not asked about dyspareunia and none reported this symptom spontaneously. The core symptoms were those considered directly caused by endometriosis, and were determined in part by the frequency with which they were reported by participants, and in part by their clinical relevance according to the advice of the consulting clinical scientific experts. Other symptoms (e.g. nausea) could be considered more ‘secondary/distal’.

Input from expert clinicians later suggested that the other symptoms identified were secondary symptoms of endometriosis as they are not necessarily directly caused by the endometriosis tissue but can be closely related to ERP and other core symptoms. Most symptoms were described to occur both during and between menstrual periods for most participants, although the severity of symptoms tended to be worse during menstruation. Generally, the experience of endometriosis symptoms was similar across adults and adolescents. Although there were some small differences in symptoms between those on hormonal therapy and those who were not, no definitive conclusions could be drawn due to the small sample sizes. These key symptoms are presented with example participant quotes in Table 2.

**Table 2:** Concept elicitation interviews: overview of key symptoms and impacts reported by participants (N = 30)

Concept	Sub-concept	Example quaote (patient ID)
*Symptoms*		
Pelvic pain or cramps (including dysmenorrhea)	Pelvic pain or cramps (n = 30)	“The main symptoms I have is pelvic pain, I have a lot of pain. I'm constantly wondering if I'm going to have more pain, and, when my period starts, it gets kind of even worse” (1–01-49-C-OT)
Vaginal bleeding	Heavy menstrual bleeding (n = 29)	“I can easily go through a pack and a half of like the Always Overnights, uh, which is about 24 pads in a day. And, and usually I double up. I'll have the overnight pad with like the, the super tampons and I can't count how many of those I use, like 48 of those.” (2–02-31-C-OT)
	Non-menstrual bleeding (n = 16)	“The spotting is usually, um, like it happens at different times … and I'm nowhere near the time when it's time for my cycle.” (1–19-34-C-OT)
Dyspareunia*	Dyspareunia (n = 16)	“Having sex is extremely uncomfortable.” (3–04-37-NC-NT)
Symptoms associated with pelvic pain or cramps	Tiredness (n = 29)	“I just feel like someone's just like draining out of me, and so I just like feel really drowsy, because I'm just in like so much pain… and so it just like really takes a lot out of me.” (1–05-17-C-OT)
	Nausea (n = 26)	“It makes you feel like you sick to your stomach. You may not have to, you know, puke or vomit, but you feel like you want to.” (2–04-39-CNC-OT)
	Loss of appetite (n = 26)	“The cramping, um, it prevents me from eating… I can't eat.” (1–08-34-NC-OT)
	Hot flashes (n = 16)	“sometimes I'll get temperature… it feels like it just gets warm. Like, when I’m having the pain, the temperature rises.” (1–03-31-C-OT)
Bowel symptoms	Constipation (n = 16)	“I be constipated…every menstrual cycle.” (2–04-39-CNC-OT)
	Bowel-related pain (n = 13)	“I was getting a pain in my stomach that felt like somebody was stabbing me inside the stomach… But it was coming from my rectum all the way to the stomach… So it felt like they were stabbing me with a knife.” (1–08-34-NC-OT)
	Diarrhea (n = 12)	
		“When the cramps were really, really bad, then the bowel movements would be like literally as smooth as butter.” (3–04-37-NC-NT)
*Impacts*		
Emotional functioning	Feel frustrated (n = 23)	“I'm the only one experiencing this pain, and like the littlest things will start annoying me, just because I want to be left alone.” (1–06-17-CNC-OT)
	Feel worried (n = 17)	“I got pads, I got all kind of protection, I'm wondering about my clothes, and sometimes I keep an extra set of clothes in the car, you know, it's a constant, you know, I got to worry about it.” (1–01-49-C-NT)
	Feel sad (n = 15)	“…kind of like upsetting because I really want to hang out with them and like be able to do like what else they can do, um, like with all the active stuff they do.” (1–10-15-C-NT)
	Mood swings (n = 11)	“…one day I'll be crying and sad for no reason. The next day I'll be happy, so, yeah, it affects me a lot.” (1–08-34-NC-OT)
Physical functioning	Sport or exercise (n = 26)	“I don't work out when I have my period… I think it's the whole bleeding and cramping… and, if I do work out, it's very minimal…it's more like walking. There's no abdominal muscle movements.” (1–07-48-NC-NT)
	Difficulty walking (n = 23)	“…it's mostly the movement, like walking, and I can't do much, I'm always cautious this is going to start the pain… I don't just walk around like I used to.” (1–01-49-C-NT)
	Difficulty lifting or carrying things (n = 16)	“I don’t lift anything. I can't. I feel like I can't. I don’t have the strength.” (2–08-31-CNC-NT)
	Difficulty getting up or standing up from sitting (n = 13)	“…I went to stand up and I couldn’t the pain was so severe. Like I was sitting to standing, and then I actually had to stop and bend over and it took my breath away.” (3–04-37-NC-NT)
Social or leisure activities	Social or leisure activities (n = 24)	“…it does limit you doing things going—like I said, going shopping. Just hanging out with friends, you know, so it's just tough some days.” (3–04-37-NC-NT)
Work	Missing days off work (n = 13; 12 adults and 1 adolescent)	“…I may not be able to go to work because I didn’t get enough sleep or, or I'm really uncomfortable.” (1–13-48-CNC-NT)
Sleep disturbance	Night-time awakening (n = 17)	“it's just uncomfortable to sleep so tossing and turning a little bit more… needing to get up and change pads just in case. Checking to see if I need to change a pad… so it's less comfortable sleep.” (2–02-32-C-OT)
	Difficulty getting to sleep (n = 13)	“It's very difficult to fall asleep, because they said, since I'm always in pain… so like it messes with my sleep. That's why I sometimes take Ambien.” (1–08-34-NC-OT)
Sexual functioning*	Avoidance of sexual activity (n = 13)	“…sometimes it's painful… and I can kind of tell I guess by the way that I feel like that mid-month time that it might be painful, so I'll kind of like abstain instead of making it a big deal.” (2–03-37-C-NT)
	Reduced sexual desire or interest (n = 10)	“…it's like something I don’t want to do. I'm not like interested. You know, like because it's not really going to benefit me at all. It's going to cause me pain.” (2–08-31-CNC-NT)
School	Missing days off school (n = 12; 9 adolescents and 3 adults)	“…some days I stay home because my cramps get really bad in the morning so my mom just lets me stay home and basically sleep all day.” (1–09-12-C-NT)

^*^Dyspareunia and impacts to sexual functioning were only discussed with adult participants (N = 20)

Pelvic pain was the only symptom reported by all 30 participants, of whom 29 (96.7%) reported this spontaneously. It was also reported to be the most bothersome (n = 28/30 [93%]) and most frequently experienced symptom (n = 27/30 [90%]). Participants most commonly described ‘cramps’ (n = 12) and ‘pain’ (n = 9), although specific descriptors such as ‘sharp’ (n = 10) and ‘dull/achy’ (n = 8) were also used. Pain was located in the back/lower back (n = 13), pelvic area (n = 11), lower abdomen (n = 9), lower stomach (n = 5) and the upper legs (n = 3). For most participants, this pain was experienced both during and between menstrual periods (n = 26), while others experienced pelvic pain during menstruation only (n = 4). Table 2 presents the overall number of participants experiencing each symptom. When participants were discussing their pelvic pain, they were asked whether they experienced any other symptoms at the same time as their pelvic pain. Participants reported that they had experienced the following symptoms at the same time as their pelvic pain: nausea (n = 16), loss of appetite (n = 11), headaches (n = 8), bowel symptoms (n = 6), tiredness/fatigue (n = 6), vomiting (n = 4), dizziness/lightheadedness (n = 3), and bloating (n = 3). Of seven participants who were asked, most (n = 5) considered pelvic pain and dyspareunia to be different.

All participants discussed being impacted by their endometriosis, as outlined in Fig. 2. Notably, many of the impacts were described as directly resulting from ERP. These key impacts are further detailed with example participant quotes in Table 2. Descriptions of less frequently reported symptoms and impacts are available in Supplementary Table 1 (see Additional file 1).

### Concept saturation

All key symptoms were reported in the first two sets of interviews. Although two symptoms (‘urinary symptoms’ and ‘swelling’) were spontaneously reported for the first time in the final set of interviews, neither are considered to be typical symptoms of endometriosis based on the published literature [27] and input from clinical experts. Experiences of endometriosis across the whole sample were generally very similar, indicating that concept saturation was achieved.

### Modification of the DysDD to form the EDD

The DysDD was modified to add items assessing additional symptoms and impacts identified as most relevant to endometriosis based on the CE results presented in the conceptual model (Fig. 2). The decision to develop new items was typically determined by how many participants reported each concept (items were initially drafted for concepts reported by > 50% of the participants), the proportion of participants spontaneously reporting the concept versus those after probing, whether the concept was related to ERP, whether the concept was reported as most bothersome or most frequently occurring by participants, and whether the concept was clinically relevant (as confirmed by clinical experts). Moreover, the additional impact items align with established recommendations for assessing impacts of chronic pain in clinical trials, including the recommendation to assess emotional impacts which are often overlooked. [28, 29].

Item wording was informed by the language used in the CE interviews. Items assessing pelvic pain (including dyspareunia) were framed as ‘at its worst’ given evidence that worst pain reports tend to be more reliable than average pain reports [30]. A 0 (no pain or cramps) to 10 (extreme pain or cramps) NRS was used for pain items as this is widely accepted for capturing pain severity [31]. Response formats for other items varied depending on the question, including a 5-point verbal rating scale (VRS) implemented for most impact items (Not at all, Slightly, Moderately, Quite a bit, Extremely). ‘Not applicable’ options were included where relevant to provide a suitable option for participants who may not have engaged in an activity that day. This measurement approach is consistent with other patient-reported assessments of endometriosis impacts. [32].

The CE results supported a 24-h recall period accounting for the daily variability reported by participants in relation to their endometriosis symptoms and impacts, as well as minimising recall bias.

### Stage 2: cognitive debriefing of the EDD

The draft EDD tested in the CD interviews comprised 27 items and 6 instructions. Feedback from participants suggested that the items (including response options and 24-h recall period) were well-understood across both rounds of CD interviews. Misunderstandings tended to be in relation to two adolescents who struggled with the CD process. The draft items were generally relevant to participant experiences of endometriosis. Although some items (e.g. diarrhea) were comparatively less relevant than others, the combined CE and CD data suggested that all items were sufficiently relevant to endometriosis to warrant their retention in the EDD at this point in time, pending psychometric evaluation. Additionally, participants’ feedback generally supported item wording where a direct attribution to ERP was made. Supplementary Figure 1, Supplementary Figure 2, Supplementary Figure 3 and Supplementary Figure 4 present results demonstrating understanding and relevance for each item (see Additional file 1).

**Fig. 3:**
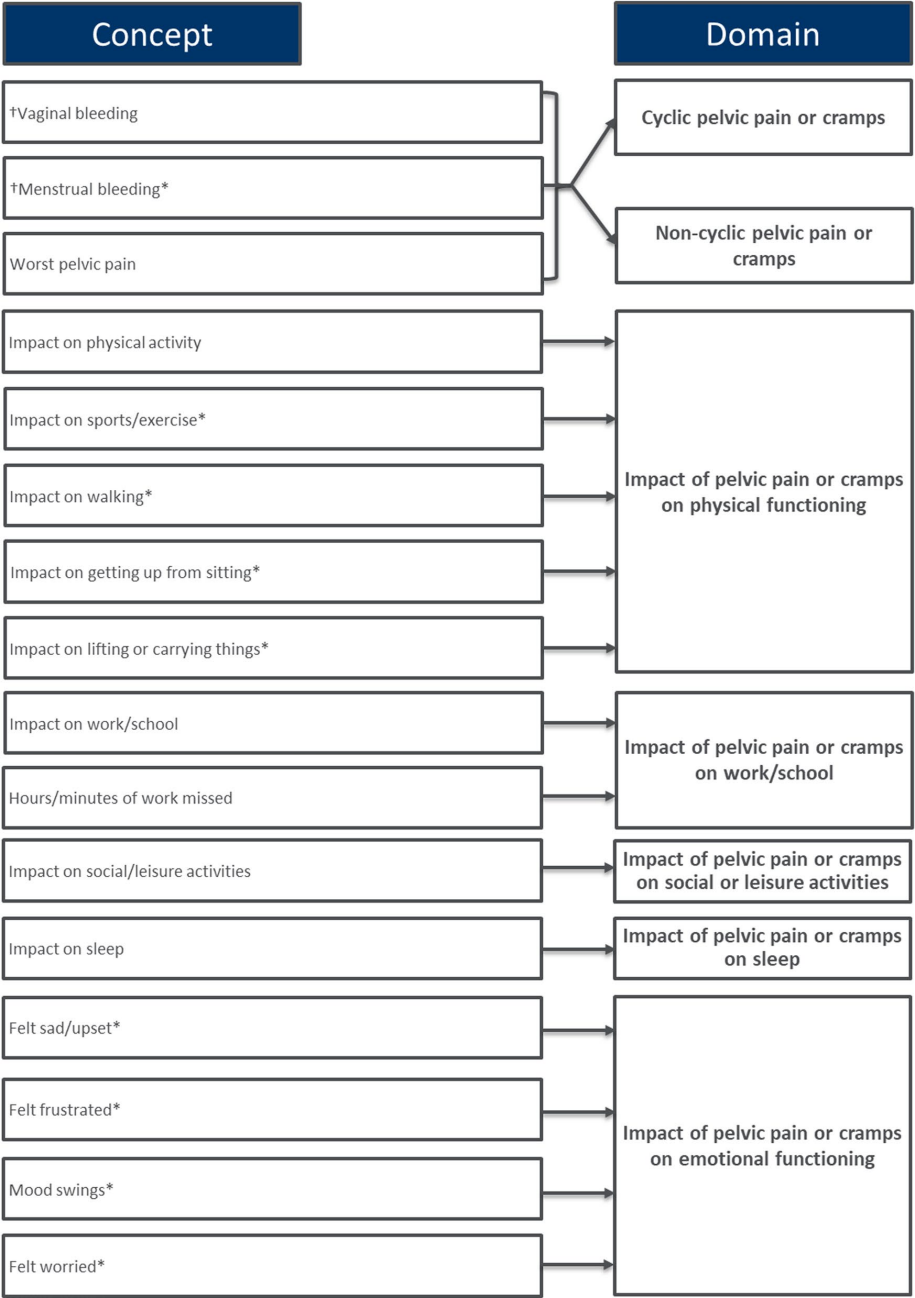

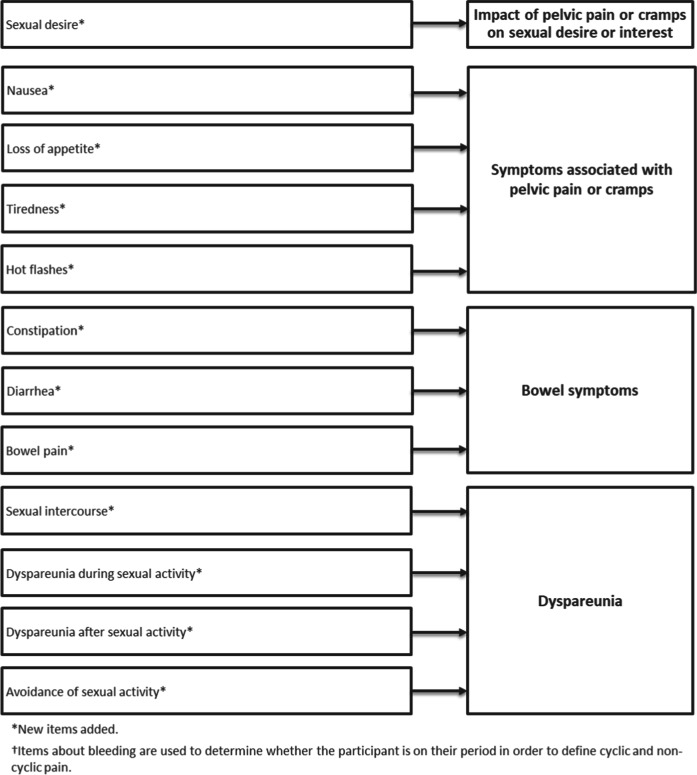
Endometriosis daily diary (EDD) conceptual framework

Between rounds, small wording updates were made to several items, including changing ‘limit you’ to ‘impact’ to improve translatability, and some sentence structures and response options were updated to ensure consistency across items. Following both rounds, all items were retained pending psychometric validation. Based on participant feedback and input from clinical experts, the item assessing dyspareunia was also separated into two items; one assessing dyspareunia during sexual intercourse/activity, and one assessing dyspareunia after sexual intercourse/activity. The final instrument (Fig. 3) includes six instruction screens and 28 items (note: the number of screens shown each day depends on responses to the items assessing vaginal bleeding, and engagement in sexual activity, due to skip patterns presenting only relevant questions based on previous responses). In Fig. 3, the 21 new items were each denoted by an asterisk.

Participants were also asked what change would be meaningful to them in relation to key symptoms and impacts of endometriosis. These results are available in the CD results: Participant-reported meaningful change (see Additional file 1).

### Stage 3: usability and feasibility testing

All participants (N = 15/15) reported that the ePRO device was easy to use and that they were able to integrate diary completion into their daily routine, with most participants estimating that it took 10 min or less (n = 13/15; 86.7%) to complete the diary. Additionally, all participants reported that the time taken to complete the diary each day was acceptable and that the diary length posed no problems to their daily completion. Suggestions for device improvements (namely the battery life of the devices) and for minimizing missing diary entries were captured and will be implemented in future studies.

## Discussion

The goal of this work was to develop a single instrument measuring symptoms and impacts related to ERP to support primary endpoints in the clinical development program of a novel ERP treatment. The instrument was intended to assess the severity of ERP in target endometriosis populations, and the degree to which the reduction of pelvic pain could benefit patients in their functioning and daily activities. The existing instruments were not appropriate in this context of use as they were not ERP specific, or were not available at the time this study was initiated. For example, the Endometriosis Health Profile- 30 (EHP-30) does not assess symptoms and its impact assessment includes some broader concepts of interest that are not directly related to ERP.

The study results add value to the endometriosis literature by presenting and confirming the detailed conceptual model of the patient experience of endometriosis and delineating patient-reported associations between ERP and other endometriosis symptoms. This conceptual model and supporting participant accounts were integral to the modification of the DysDD to form the EDD and were developed in line with recommendations for instrument modification provided in the recently released FDA Patient-Focused Drug Development Guidance [33]. Moreover, by adhering to PRO guidelines [12, 18] and best practice at each stage of development [10], the resulting EDD demonstrates good content validity per regulatory guidance, and will be suitable for translation into other languages while retaining conceptual equivalence.

Incorporating both patient and clinical expert feedback at key stages of PRO development and testing is vital to ensure that the resulting instrument is robust and suitable for administration in multiple contexts, including supporting clinical trial endpoints. The next steps of this research are to evaluate the psychometric properties of the EDD, define scoring, and estimate meaningful change thresholds and responder definitions. Item performance should also be assessed both individually and collectively, and any redundant or poor-performing items considered for deletion. However, such decisions should also take account of the qualitative findings and clinical importance of concepts.

One limitation that all interviews were performed with patients in the US. However, similar research conducted across the US, Germany, and France [14] did not find differences in patient experiences of endometriosis across countries nor in terms of the language used to describe their experiences. As noted elsewhere, the unmet need for a robust and rigorously developed PRO assessing symptoms and impacts of ERP has been addressed concurrently by different researchers and sponsors, and several other similar PROs have recently been published [14, 15, 34]. However, to see multiple concerted efforts addressing what was previously an under-researched area is a great step forward for endometriosis research. Furthermore, there is substantial value in recognising consistencies across qualitative research projects and the resulting knowledge and understanding of the patient experience of endometriosis that is now available. Indeed, it is increasingly recognized within the PRO field that ‘Core Outcome Sets’ for a specific condition or context of use are established by defining key concepts that should be consistently measured in all trials and where possible identify measures that provide adequate measurement of those concepts [35, 36]. Recent research conducted in accordance with Core Outcome Measures in Effectiveness Trials (COMET) recommendations [37] has provided some preliminary recommendations for Core Outcome Sets for endometriosis, with pain and impacts to quality of life at the foundation of the set [38]. Future research will ideally corroborate and add to this set as necessary. While it would be ideal to have a single gold standard measure of endometriosis, instruments are not always publicly available at the time they are required, and it is reassuring that the EDD and similar measures are consistent in prioritising assessment of vaginal bleeding, both cyclic and non-cyclic pelvic pain, and dyspareunia.

Beyond these core symptoms, the EDD differs to similar measures principally by including all items within a single diary intended to be administered daily with a 24-h recall period, reducing potential recall bias. This allows for the fact that the symptoms, and related impacts, of ERP fluctuate daily. While a longer recall period was considered for some of the impact items (e.g., impact on “taking part in sports or exercise”), it was discussed and agreed with clinical scientific experts that all items should be asked with a 24-h recall for consistency and accuracy. As an effort to reduce patient burden, for the eight items reflecting activities that may not occur on a given day (e.g., “take part in sports or exercise”), a response option of “Not Applicable” has been included. Concerns of participant burden have also been initially addressed by the results of Stage 3, which demonstrated the feasibility of this format of administration, meaning that the EDD can be confidently used to assess the symptoms and impacts of ERP on a daily basis. However, it is recognized that usability/feasibility test did not mimic clinical trials. The EDD development thus far remains patient-focused by including items assessing a range of concepts identified as relevant to patient experiences. The next phase of development will be to confirm and assess psychometric properties in a clinical trial. It is expected that the length of the EDD will be reduced following psychometric testing wherein items of low relevance, redundancy, or poor performance will be identified, and streamlining the instrument will be a priority in such analyses. Currently, skip patterns can be implemented to ensure that patients answer questions relevant to their experience that day based on their responses, and psychometric validation will further inform the utility of skip patterns to streamline EDD administration and minimise patient burden.

## Conclusion

An existing PRO (the DysDD) was successfully modified and formed the basis for the development of a content-valid instrument for daily assessment of ERP symptoms and impacts (the EDD). Best practice scientific standards were followed both throughout the initial DysDD development and the modification to form the EDD, the result of which is a robust instrument with good content validity for both adults and adolescents with endometriosis. Additional testing will evaluate the psychometric properties of the EDD, which is expected to be a reliable and valid PRO for use in ERP clinical studies.

## Supplementary Information

**Additional file 1.** **Supplementary materials.**CE results: Additional symptoms and impacts reported during CE interviews. **Supplementary Table 1.** Concept elicitation interviews: overview of additional symptoms and impacts reported by participants (N = 30). CD results: Figures demonstrating understanding and relevance for EDD items. **Supplementary Figure 1.** Understanding of each EDD item in Round 1 of CD interviews. **Supplementary Figure 2.** Relevance for each EDD item in Round 1 of CD interviews. **Supplementary Figure 3.** Understanding of each EDD item in Round 2 of CD interviews. **Supplementary Figure 4.** Relevance for each EDD item in Round 2 of CD interviews. CD results: Participant-reported meaningful change.

## Data Availability

Data sharing is not applicable to this article as no quantitative datasets were generated or analysed during the current study.
