# Supplementary material for: The endometriosis daily diary: qualitative research to explore the patient experience of endometriosis and inform the development of a patient-reported outcome (PRO) for endometriosis-related pain
Source: J Patient Rep Outcomes. 2022 Jan 15;6:5. doi: 10.1186/s41687-021-00409-8 (PMC8761214; doi:10.1186/s41687-021-00409-8)
Supplement: Supplementary file 1 — Additional file 1. Supplementary materials.CE results: Additional symptoms and impacts reported during CE interviews. Supplementary Table 1. Concept elicitation interviews: overview of additional symptoms and impacts reported by participants (N = 30). CD results: Figures demonstrating understanding and relevance for EDD items. Supplementary Figure 1. Understanding of each EDD item in Round 1 of CD interviews. Supplementary Figure 2. Relevance for each EDD item in Round 1 of CD interviews. Supplementary Figure 3. Understanding of each EDD item in Round 2 of CD interviews. Supplementary Figure 4. Relevance for each EDD item in Round 2 of CD interviews. CD results: Participant-reported meaningful change. [file 41687_2021_409_MOESM1_ESM.docx]

## Supplementary materials

### CE results: Additional symptoms and impacts reported during CE interviews

| **Supplementary Table 1. Concept elicitation interviews: overview of additional symptoms and impacts reported by participants (N=30)** | | |
| --- | --- | --- |
| Concept | Sub-concept | Example quote (Patient ID) |
| **Symptoms** | | |
| **Additional symptoms** | Headache (n=17) | *“I experience headaches when I'm off and on my period… my head will start like banging really hard.”* (1-04-12-C-NT) |
|  | Discharge (n=15) | *“ I’ll get like, um, like white sticky like discharge and, um, usually in the beginning of my period and like before my period.”* (1-10-15-C-NT) |
|  | Tender or sensitive breasts (n=13) | *“Your breasts, they feel swollen—they might be swell. They hurt real bad. They feel real tender to the touch, so you don’t really want to touch them.”* (2-04-39-CNC-OT) |
|  | Skin changes (n=13) | *“Sometimes it gets drier and it gets more itchy.”* (2-03-37-C-NT) |
|  | Dizziness/ lightheadedness (n=12) | *“I start getting really dizzy and my head, like I just feel like I'm about to pass out.”* (2-09-17-CNC-NT) |
| **Impacts** | | |
| **Emotional functioning** | Withdrawn (n=10) | *“I feel very withdrawn… especially when I'm on my cycle, it just seems like it's the worst time… If there's events that's going on, the first thing I say is, well if it's between this time and this time in the month, then, you know, I don’t want to agree to come.”* (2-06-33-NC-NT) |
| **Physical functioning** | Staying in bed (n=16) | *“…you got a headache and you bleeding extremely heavy and you got pain all on your body and you're not going to want to do nothing. Like I said, I don’t want to get out the bed. I just want to just lie down the whole time.”* (2-08-31-CNC-NT) |
|  | Avoiding movement (n=11) | *“…a bad day is I don't even want to get out of bed. I’m in so much pain… constantly cramping, I’m constantly having a lot of stomach issues, stomach pains. I don't want to eat, I don't like want to move.”* (1-11-29-C-OT) |
| **Social or leisure activities** | Avoid socializing/going out (n=12) | *“I don't go out. If there’s a get-together, I just don't go… I don't want to have to explain why I’m not feeling well. I don't want to see everybody else having a good time."* (1-11-29-C-OT) |
|  | Relationships with friends (n=11) | *“Well, um, when they want to hang out and I'm like going through that pain, I don’t really want to at the time. And I feel very like negative when I'm having these pains and it's actually—like my friends have noticed it and it affects them, and then I—it affects me as a person 'cause I'm like, I don’t want to treat my friends like that but I'm going through this and I don’t think they understand it.”* (2-09-17-CNC-NT) |
|  | Relationships with family (n=1) | *“I think it's impacted my relationships with my sisters and my like family, because they don't understand, so, when I'm always in pain, I'm always at home… I won't answer my phone for days, then they get mad. They know I'm sick, but they get mad that I don’t answer, so I think it's affected the relationship… Now we don't talk every day like we used to.”* (1-08-34-NC-OT) |
| **Work** | Productivity (n=10) | *“It makes me a little less productive, but I try to still keep on going and do what I need to do…. Just having to go to the bathroom more often, feeling that pain, like, ugh… no matter what position you're in.”* (1-03-31-C-OT) |
| **Activities of daily living** | Housework/chores (n=20) | *“Too much bending and, and lifting. I definitely can't do that… sweeping, mopping, bending down, uh, cooking. Just 'cause it requires you to be like on your—you know, up on your feet doing things. I don’t have, I don't have the energy"* (2-08-31-CNC-NT) |
|  | Shopping/errands (n=16) | *"…if I'm in a lot of pain and I'm bleeding a lot, I won't shop and I won't run any errands... I'll typically do it when I'm feeling much better"* (2-06-33-NC-NT). |
|  | Clothing (n=16) | *“Because I have to always worry about like bleeding through and like going places, especially in like the summer, I have to carry on extra clothes, like swimming and school is awful.”* (1-06-17-CNC-OT) |
|  | Need to rest/nap (n=11) | *“if [the pain] gets too bad, then I'll rest. I'll sit down or I'll go somewhere where I can try to elevate my feet a little bit… but if it gets too bad, then I, I have to lie down... So it definitely has its hold on my everyday life.”* (2-06-33-NC-NT) |
|  | Hobbies (n=10) | *“I do theater. I sometimes have to stop from that… I've missed it a lot actually. Um, I've even missed like auditions before and, um, just rehearsals and stuff.”* (2-09-17-CNC-NT) |
|  | Generally doing less (n=10) | *“I just do everything in consideration, so like in like less—I just do less of everything.”* (1-05-17-C-OT) |

### CD results: Figures demonstrating understanding and relevance for EDD items

Supplementary Figure 1. Understanding of each EDD item in Round 1 of CD interviews

Supplementary Figure 2. Relevance for each EDD item in Round 1 of CD interviews

Supplementary Figure 3. Understanding of each EDD item in Round 2 of CD interviews

Supplementary Figure 4. Relevance for each EDD item in Round 2 of CD interviews

### CD results: Participant-reported meaningful change

Across both rounds of CD interviewing, participants were also asked which level of change would be important to them in relation to pelvic pain at its worst, pelvic pain during sexual intercourse or activity at its worst, bowel-related pain, nausea, sports and exercise, walking, lifting and carrying, and getting up from sitting. Levels of important change were described using the response scales provided for each item. Most participants asked reported that a 1-point change in general pelvic pain at its worst on the 0-10 NRS would not be an important change (n=7/9). Participants provided a diverse range of suggestions of what level of change *could* be considered important, however a 3-point change received the most support. In terms of dyspareunia, there was less consensus among participants as to what would constitute an important change, however there was most support for a 3-point (n=2) or 4-point (n=3) change on the 0-10 NRS. Regarding bowel-related pain, feedback was mixed, with evidence that a 1-point change (n=5), a 3-point change (n=6), or a 4-point change (n=6) on the 0-10 NRS could all be important to participants. For nausea the possible levels of change were based on the 0-4 VRS (0 not at all - 4 extremely), and several participants reported that a 1-point change would be important (n=11), with others suggesting that it would need to be a 2-point change to be important (n=4).

In terms of impacts, important change was reported in relation to the 0-4 VRS for each item. For difficulty taking part in sports/exercise, a 1-point (n=6) or 2-point (n=7) change were most frequently endorsed as important, although one participant reported that a 3-point change (n=1) would be required to be considered an important change. In terms of difficulty walking, there was strong support for a 2-point change (n=10), however some participants did report that even a 1-point change would be meaningful (n=3). Similarly, a 2-point change in difficulty lifting/carrying received the most support (n=7), although a 1-point change would also be important to some (n=5). For difficulty getting up from sitting, there was a strong level of consensus that a 1-point change would be important (n=10), however a 2-point change was also reported by some (n=3). However, due to the small number of participants, caution should be employed when drawing conclusions from these results, which cannot be generalized beyond the participants in this research.
